# Probing Kinetics Beyond Tafel Unlocks Highly Accurate Exchange Currents for Aqueous Zinc Metal Batteries

**DOI:** 10.1002/smtd.202500508

**Published:** 2025-04-24

**Authors:** Ashutosh Rana, Md. Arif Faisal, Jeffrey Edward Dick

**Affiliations:** ^1^ Department of Chemistry Purdue University West Lafayette IN 47907 USA; ^2^ Elmore Family School of Electrical and Computer Engineering Purdue University West Lafayette IN 47907 USA

**Keywords:** aqueous zinc metal batteries, electrodeposition, electron‐transfer kinetics, fast‐scan voltammetry, mass transfer, rate‐limiting step, ultramicroelectrodes

## Abstract

Understanding the kinetics of zinc electrodeposition on current collectors is crucial for improving aqueous zinc‐metal battery (AZMB) performance, yet it remains largely unexplored. A major challenge within the field is the inconsistent reporting of kinetic parameters, particularly the exchange current density (*j*_0_), which is essential for accurately modeling and simulating zinc electrodeposition reactions. In this study, fast scan voltammetry on tungsten ultramicroelectrodes (UMEs) is employed to decouple mass transfer effects and isolate charge transfer kinetics. These results show that while zinc electrodeposition is a two‐electron process, the rate‐limiting step involves a one‐electron transfer, validated through Butler‐Volmer and Marcus‐Hush models. This work also identifies significant limitations of Tafel analysis for certain zinc electrolyte systems, as their kinetically quasi‐reversible/irreversible nature prevents the existence of a true Tafel regime (±118 mV kinetic regime). For such systems, the Allen‐Hickling approach is proposed as a more accurate method for probing zinc electrodeposition kinetics. We report *j*_0_ values for ZnSO_4_ (0.20 *A*/*cm* ^2^), Zn(OTf)_2_ (0.42 *A*/*cm* ^2^), and ZnCl_2_ (0.46 *A*/*cm* ^2^) and provide clear guidelines for precise kinetic analysis based on the width of the kinetic regime. Finally, the impact of accurate kinetic parameter measurements on coin cell performance is demonstrated, revealing that lower accurately determined *j*_0_ values correlate with improved long‐term cycling stability, following the trend ZnSO_4_ > Zn(OTf)_2_ > ZnCl_2_, aligning with predictions from Sands’ model. This work provides the first systematic kinetic investigation of counter anions in aqueous zinc‐metal batteries, offering critical insights into how electrolyte composition influences charge transfer kinetics. These findings advance our fundamental understanding of AZMB kinetics and offer a framework for optimizing electrolyte compositions and electrode designs to enhance battery performance and durability.

## Graphical abstract

Ultramicroelectrodes are employed to investigate the kinetics of zinc electrodeposition for commonly used electrolytes—Zn(OTf)₂, ZnCl₂, and ZnSO₄. The suitability of kinetic analysis techniques such as Tafel analysis, Butler‐Volmer, Marcus‐Hush, and Allen‐Hickling depends on the kinetic reversibility of the system. Accurately measured exchange current densities can serve as reliable descriptors for anode stability in zinc batteries.

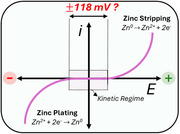

## 1 Introduction

The urgent need for alternative battery chemistries, distinct from conventional lithium‐ion technologies, is driven by several critical factors that are reshaping the landscape of energy storage solutions. While lithium‐ion batteries have achieved remarkable efficiency and widespread adoption, they face significant challenges. This includes limited availability of lithium resources, safety concerns related to thermal runaway, and the environmental impact associated with resource extraction and disposal. In contrast, aqueous zinc‐metal batteries (AZMBs) present promising alternatives that address these issues by utilizing abundant, non‐toxic materials, enhancing safety through the elimination of flammable electrolytes, and significantly reducing the overall environmental footprint.^[^ ^1^, ^2^, ^3^ ^]^ As the development of AZMBs accelerates, a comprehensive understanding of the factors influencing the kinetics of electron transfer at the zinc electrode/ anode surface is essential. Specifically, the reaction, *Zn* ^2 +^ + 2*e* ^−^ →  *Zn* ^0^ is fundamental to the efficiency and performance of these systems.^[^ ^4^, ^5^ ^]^ However, the rapid advancement of AZMBs is currently impeded by critical challenges, including dendrite formation during zinc electrodeposition, corrosion of the zinc metal anode, hydrogen evolution reaction during zinc plating, and various side reactions that compromise battery performance.^[^ ^6^, ^7^, ^8^, ^9^, ^10^ ^]^ To address these challenges, several strategies are employed, such as the introduction of additives, co‐solvents, modifications to current collectors, and enhancements to separators.^[^ ^11^, ^12^, ^13^, ^14^ ^]^ Across all these domains, an understanding of the kinetics of electrodeposition is crucial, both prior to and following the implementation of these modification strategies.^[^ ^4^, ^5^, ^15^ ^]^ Achieving a stable anode for AZMBs requires a uniform, dense, and dendrite‐free morphology, which is intricately linked to the nature of the solid‐electrolyte interphase (SEI) that forms during the deposition process.^[^ ^16^, ^17^, ^18^, ^19^, ^20^, ^21^ ^]^ The morphology of the electrodeposited metal is primarily influenced by two key factors: charge transfer kinetics, characterized by the two‐electron transfer reaction *Zn* ^2 +^ + 2*e* ^−^ →  *Zn* ^0^, and the mass transfer/ diffusion of Zn^2^⁺ ions to the electrode surface.^[^ ^4^ ^]^ In this work, we specifically focus on elucidating the intricacies of the two‐electron charge transfer kinetics, which play a pivotal role in optimizing the performance and longevity of aqueous zinc‐metal batteries.

Accurate determination of kinetic parameters such as the exchange current density (*j*_0_) and charge transfer coefficient (α) is crucial for predictive modeling and simulation of zinc electrodeposition kinetics. These parameters play a key role in refining continuum‐scale models and atomistic simulations that seek to describe nucleation, growth, and morphology evolution in metal anodes.^[^ ^22^, ^23^, ^24^, ^25^, ^26^, ^27^, ^28^, ^29^, ^30^ ^]^ Inaccurate values of *j*_0_ can lead to misleading interpretations of zinc deposition behavior, particularly in simulations that guide the optimization of electrolyte composition and current collector designs. Despite the widespread use of macroelectrodes for kinetic studies, they inherently suffer from convolution of mass transfer effects that obscure true kinetic control, leading to systematic errors in *j*_0_ determination. In contrast, ultramicroelectrodes (UMEs) provide a significant advantage by operating in a regime where diffusion is hemispherical, ensuring steady‐state conditions that effectively decouple mass transfer contributions.^[^ ^4^, ^15^, ^31^ ^]^ By leveraging UMEs and fast scan voltammetry, this study establishes best practices for obtaining highly accurate exchange current values, which are critical for validating electrochemical models and advancing the rational design of zinc‐based battery systems.

In this study, we build on our previously reported findings regarding the utilization of UMEs and fast scan voltammetry to probe the kinetics of zinc electrodeposition.^[^ ^5^ ^]^ Our earlier investigations revealed that the scan rate significantly influences crucial processes such as nucleation and growth during the deposition of Zn^2^⁺ ions, demonstrating a strong dependence of the measured exchange current on the scan rate.^[^ ^4^ ^]^ To accurately investigate charge transfer kinetics, it is imperative to employ fast scan voltammetry, as it effectively decouples mass transfer effects, ensuring that the measured current is both independent of the scan rate and reflective of true kinetic control of the electrodeposition reaction. In this work, we focus on the current transients observed in the kinetically controlled regime, emphasizing the critical observation that, despite zinc electrodeposition being conventionally described as a two‐electron process, the kinetic regime predominantly reports on the rate‐limiting step, which is a one‐electron transfer.^[^ ^31^, ^32^ ^]^ This essential concept of a rate‐limiting step is frequently overlooked and has not been thoroughly examined within the aqueous battery community. Furthermore, we delve into the intricacies of applying the widely used Tafel analysis to elucidate electron‐transfer kinetics. Our analysis reveals that a true Tafel regime is not established for zinc electrodeposition for certain electrolyte systems, primarily due to the quasi‐reversible kinetics of the electrodeposition process, which leads to significant influence of mass transfer in Tafel regime. To address these limitations, we present an alternative analytical approach utilizing Allen‐Hickling plots, which provide a more reliable framework for accurately probing electron transfer kinetics in these systems as well as systems where a true Tafel regime exists.^[^ ^33^, ^34^ ^]^ While the methodology presented in this work employs voltammetry, whereas practical AZMB cycling is conducted under galvanostatic mode, the intricacies of the kinetic processes cannot be effectively realized or studied using galvanostatic experiments alone. Voltammetry, as demonstrated here, is essential for probing these processes. Moreover, to bridge the gap, the findings of the work highlight a strong correlation between kinetic processes and stability under galvanostatic conditions, making the insights directly relevant to the long‐term cycling performance of coin cells in real‐world applications.

Beyond establishing a robust methodology for exchange current determination, we demonstrate the broad applicability of our approach across three different commonly used zinc electrolytes—ZnCl_2_, Zn(OTf)_2_, and ZnSO_4_—each containing distinct counter anions. The consistency of our results highlights the universality of the Allen‐Hickling method for obtaining accurate kinetic parameters. Importantly, we identify a clear trend in the measured exchange current values across these electrolytes, which, according to Sands' model,^[^ ^35^ ^]^ provides predictive insights into their battery cycling performance. Previous findings indicate that electrolytes with lower exchange current values exhibit higher coulombic efficiency (CE), as reduced charge transfer kinetics suppress side reactions such as hydrogen evolution and unwanted parasitic reactions.^[^ ^4^, ^36^, ^37^, ^38^, ^39^, ^40^ ^]^ To validate this prediction, we provide coin cell performance data alongside CE measurements obtained using the Aurbach protocol,^[^ ^41^ ^]^ confirming that the electrolyte with the lowest accurately measured exchange current yields the highest CE. Notably, this work represents the first systematic investigation of counter anions in the field of AZMBs from a kinetic perspective, offering unprecedented insights into how counter anion identity influences fundamental charge transfer processes. By linking electrochemical kinetics to practical battery performance, our findings establish a new framework for electrolyte selection and optimization, paving the way for more efficient and durable energy storage systems.

## 2 Results and Discussion

We first illustrate the existence of a rate‐limiting step in the zinc electrodeposition reaction, demonstrating that, despite zinc electrodeposition being a two‐electron transfer process, the rate‐limiting step involves the transfer of only a single electron. Subsequently, we establish that zinc electrodeposition can exhibit a quasi‐reversible kinetic nature for certain electrolyte systems, making traditional kinetic analysis using Tafel plots suboptimal, as Tafel analysis is more suited for systems exhibiting truly irreversible behavior. To address this limitation, we propose the application of the Allen‐Hickling methodology, which accounts for the quasi‐reversible nature of zinc electrodeposition, providing a more robust framework for kinetic evaluation across all systems irrespective of the nature of the kinetic regime (both quasi‐reversible and irreversible systems).

### 2.1 For Zinc Electrodeposition Kinetic Analysis, *n* equals one. Always

**Figure 1:**
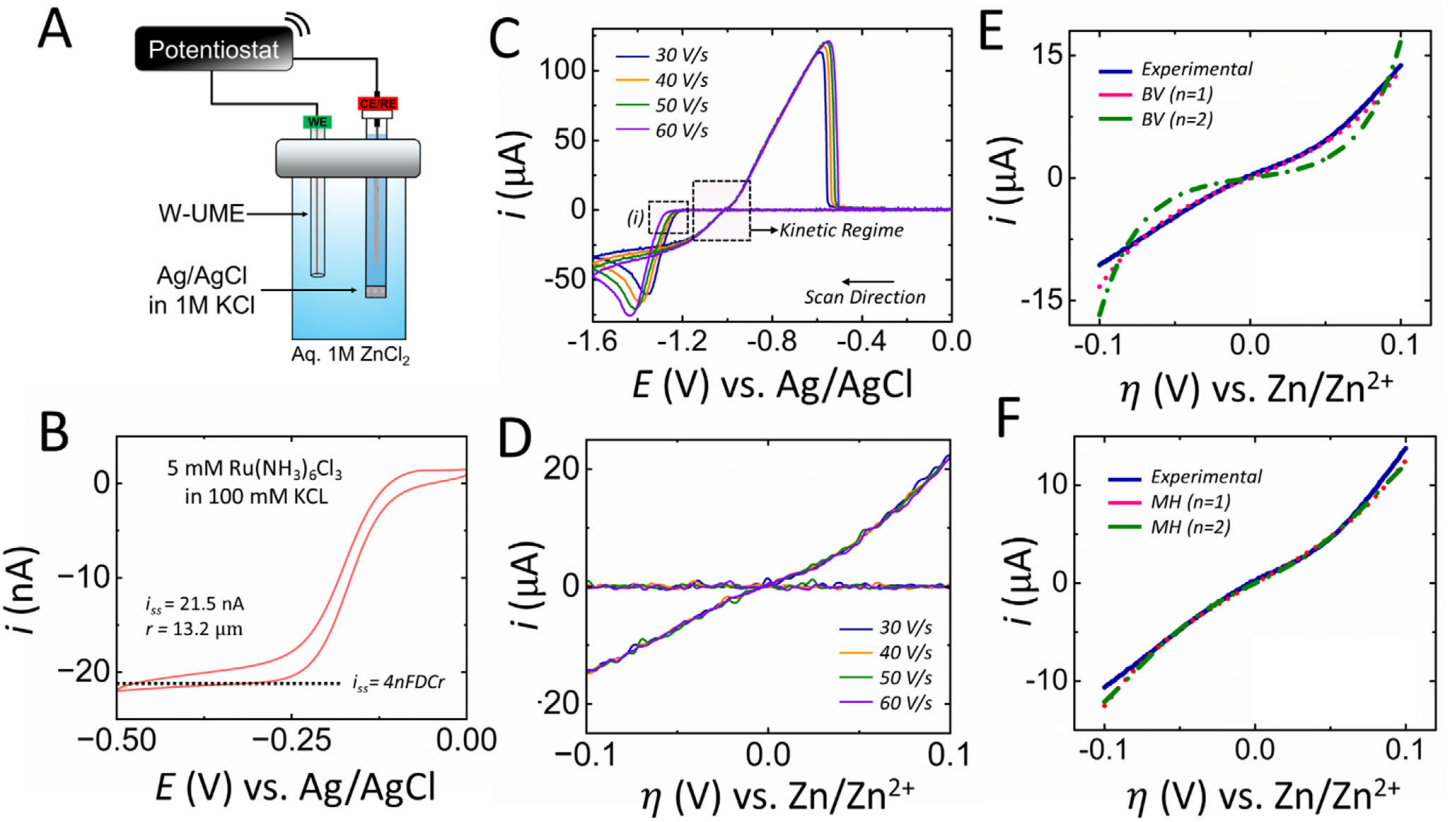
a) Schematic representation of the electrochemical setup for the fast scan cyclic voltammogram analyses with a two‐electrode setup utilizing a tungsten ultramicroelectrode as working electrode and Ag/AgCl in 1 M KCl as the reference electrode. b) Cyclic voltammogram of the tungsten ultramicroelectrode in 5 mM aqueous hexaammineruthenium (III) chloride solution in 100 mM KCl at a scan rate of 0.01 Vs^−1^. c) Fast‐scan cyclic voltammograms recorded at a scan rate of 30, 40, 50, and 60 Vs^−1^. d) Kinetic regime showing the area of the voltammograms unperturbed by mass‐transfer effects. e) The kinetic regime of fast‐scan cyclic voltammogram fitted using Butler‐Volmer formulation, where the number of electrons transferred is either one or two. f) The kinetic regime of fast‐scan cyclic voltammogram fitted using Marcus‐Hush formulation, where the number of electrons transferred is either one or two. The adjustable fitting parameters for e) and f) are discussed in the main text.

We use tungsten UMEs to investigate the electrodeposition kinetics of zinc. The UMEs were fabricated following the detailed protocol from our previous work. Zinc electrodeposition was studied using a two‐electrode setup with a W UME as the working electrode and an Ag/AgCl electrode serving as both the counter and reference electrode in UME in a 1 M ZnCl_2_/ ZnSO_4_/ Zn(OTf)_2_, electrolyte, as shown in **Figure** **1**a. A two‐electrode setup was chosen over the conventional three‐electrode configuration due to the small currents involved, allowing the reference electrode to function as a counter electrode as well.^[^ ^31^, ^42^ ^]^ Before conducting electrodeposition studies, the UME was characterized in a solution of 5 mM Ru(NH_3_)_6_Cl_3_ with 100 mM KCl as the supporting electrolyte. This characterization step is crucial for assessing the electrode size and stray capacitance. The cyclic voltammogram recorded for this characterization (Figure 1b) exhibits a characteristic sigmoidal shape of an UME. From the steady‐state current, the electrode radius was accurately determined to be 13.2 µm and accordingly, the geometric electroactive surface area (GESA) was measured to be 5.47 × 10^−6^ cm^2^. The GESA value will be used to calculate current densities throughout the work by normalizing the measured absolute current values with the GESA value. The well‐characterized electrode shown in Figure 1b was used for all the experiments discussed in this work, ensuring consistency in measurements. This standardization is crucial, especially for accurately comparing current densities across different electrolyte systems.

In our previous studies, we established the significance of employing fast scan voltammetry on UMEs to investigate the kinetics of electrodeposition.^[^ ^4^, ^5^ ^]^ This approach effectively decouples the influence of mass transfer at high scan rates, enabling us to obtain kinetic regimes in voltammograms that are solely governed by charge transfer kinetics. Figure 1c displays typical cyclic voltammograms recorded in the fast scan regime, which exceeds 10 Vs^−1^, using a W UME in a 1 M ZnCl_2_ electrolyte. We present cyclic voltammograms in the range of 30–60 Vs^−1^ obtained with a two‐electrode setup, utilizing a W UME and an Ag/AgCl counter/reference electrode. During the cathodic scan, zinc nucleation is observed between −1.2 and −1.3 V vs. Ag/AgCl, as indicated in Box 1 of Figure 1c. This is followed by a “diffusion‐limited”; peak. In the anodic scan, we observe a crossover from the reduction of zinc ions to the oxidation of electrodeposited zinc at ∼−1 V vs. Ag/AgCl. This crossover is subsequently followed by a stripping peak, after which the current returns to the baseline, confirming the complete stripping of the electrodeposited zinc of the W UME. A closer examination of the fast scan voltammograms near the crossover region reveals that the current transients overlap, regardless of the scan rates. This indicates that the measured current is unaffected by mass transfer effects and is solely controlled by kinetic processes. Consequently, the kinetic regime outlined in Figure 1c enables us to accurately determine the true kinetic parameters by directly fitting the kinetic models of electron transfer. A zoomed‐in image of the kinetic regime is shown in Figure 1d. We have previously demonstrated that this true kinetic regime is not observed at low scan rates, as the current near the crossover potential varies with changes in scan rate, indicating a significant influence of mass transfer effects. Now that we have identified the kinetic regime, we set out to answer a very fundamental question: How many electrons are transferred in the rate‐limiting step for zinc metal electrodeposition?

The structure of the electrolyte matrix in mildly acidic electrolytes, such as 1 M ZnCl_2_, ZnSO_4_, or Zn(OTf)_2_, is characterized by the presence of solvated Zn^2^⁺ ions, which primarily adopt an octahedral geometry due to coordination with water molecules.^[^ ^43^ ^]^ The electrodeposition of zinc ions from these electrolyte solutions proceeds via a “two‐electron” transfer mechanism at the electrode surface, represented by the reaction Zn^2^⁺ (electrolyte) + 2e⁻ → Zn⁰ (electrode). In this context, “electrolyte” and “electrode” denote the spatial positions of the species: Zn^2^⁺ in the electrolyte phase and Zn⁰ on the electrode surface. Although this reaction framework provides a general understanding, it can be misleading when performing kinetic analyses of zinc metal electrodeposition. The assumption that two electrons tunnel simultaneously through the interface is not physically realistic, as electron transfer in electrochemical systems typically occurs in a stepwise rather than a concerted manner. While simultaneous electron transfer can be valid for highly conjugated redox‐active molecules,^[^ ^31^, ^44^ ^]^ it is unlikely to apply to zinc electrodeposition. Consequently, it is imperative to identify the true rate‐limiting step to accurately elucidate the electron transfer kinetics associated with zinc metal deposition.

To validate the hypothesis of a rate‐limiting step involving a single electron transfer, we fitted the experimental data using the current‐overpotential equation, a modified form of Butler‐Volmer equation under no mass transfer effects, as shown in Equation (1). This equation accounts for how the rate of electron transfer at the electrode interface depends on the applied potential, the number of electrons transferred, and the activation energy/ overpotential of the reaction under no mass‐transfer effects, i.e., in the kinetic regime of fast‐scan cyclic voltammograms identified in Figure 1.

(1) $$i=i_{0}\left(e^{\left(1-\alpha \right)f\eta }-e^{-\alpha f\eta }\right)$$

In the above equation, *i* is the total current at the working electrode, *i*_0_ is the exchange current, α is the transfer coefficient, η is the overpotential and *f* is *nF*/*RT* (*n* is the number of electrons transferred (1 or 2), *F* is the Faraday's constant (98 500 C mol^−1^), *R* is the gas constant (8.314 J mol^−1^), and *T* is the temperature (298 K). To fit the data to Equation (1), we assumed that the crossover potential (−1 V vs. Ag/AgCl) represented the equilibrium potential. This assumption allowed us to convert the potential on the x‐axis, as shown in Figure 1c, to an overpotential (η) versus Zn/Zn^2^⁺, as depicted in Figure 1d. We initially fitted Equation 1 in a classical manner, applying a constant value of α as 0.5 and *n* as either 1 or 2. A least square fitting was performed, treating *i*_0_ as the fitting parameter. Note that the fitting can only be perferomed in the kinetically controlled region, ideally in a small overpotential range (100 mVs) around the crossover potential. The results of this fit are presented in Figure 1e. Based on the fit shown in Figure 1e, it is evident that using a value of *n* equals 2 leads to a significant deviation from the experimental results compared to using *n* equals 1. This provides strong evidence for the hypothesis that the rate‐limiting step involves the transfer of only one electron. It is also important to recognize that the kinetic regime depicted in Figure 1c reflects only the rate‐limiting step and not the overall two electron transfer zinc electrodeposition reaction. Prior research in the field of lithium electrodeposition has demonstrated the applicability of more complex models, such as the Marcus‐Hush (MH) model,^[^ ^5^, ^15^ ^]^ which is conceptually similar to Equation (1) but allows for potential dependence of α, as expressed in the following relationship (Equation 2). The Marcus‐Hush model extends the Butler‐Volmer framework by explicitly considering the role of solvent reorganization energy in electron transfer (λ) by assuming a potential dependent transfer coefficient (α). The value of λ reflect the kinetics of molecular reorganization during zinc ion solvation, where a higher value indicates slower desolvation kinetics prior to electrodeposition.

(2) $$\alpha =\frac{1}{2}+\frac{e\eta }{4\lambda }$$

In Equation 2, *e* represents the charge of an electron and λ denotes the solvent reorganization energy. The other variables denote parameters discussed above. In this case, we performed the least square fitting for both *n* equals 1 and 2, but with two fitting parameters (*i*_0_, λ). The results are presented in Figure 1e. In this case, we found that both scenarios yielded similar fits, which is not surprising given the introduction of two fitting parameters into the system. However, the values of these fitting parameters do provide important insights, which are discussed in the following section (see **Table** **1** for values of fitting parameters, where *i*_0_ has been converted to *j*_0_ to show current density).

**Table 1:** Fitting parameters obtained using Butler‐Volmer and Marcus‐Hush formulations for electron transfer kinetics.

No of electrons transferred	Exchange Current Density, *j*_0_ (*A*/*cm* ^2^) (BV model)	Exchange Current Density, *j*_0_ (*A*/*cm* ^2^) (MH model)	Solvent Reorganization Energy, λ (eV)
*n* = 1	0.35	0.38	0.53
*n* = 2	0.06	0.16	0.14

Table 1 presented above reveals important findings. First, for *n* equals 1, the exchange current density values are similar for both the BV and MH based fittings, with the MH model indicating a λ value of 0.53 eV. In contrast, using *n* equals 2 results in significantly greater differences between the exchange current values for the BV and MH models, with the MH model showing a λ value of only 0.14 eV. Another crucial aspect is the significant difference in the value of λ. The low value of λ for *n* equals 2 in the MH model seems too small for the electrodeposition of a divalent ion like Zn^2+^.^[^ ^15^, ^45^ ^]^ Overall, these findings in conjunction with existing literature supports the conclusion that using *n* equals 1 is the appropriate choice for the kinetic analysis of zinc electrodeposition.

Prior research by Kim and Jorne et al. in the 1980s demonstrated that the reaction order for the cathodic electrodeposition of zinc is unity.^[^ ^32^ ^]^ The kinetic mechanism for zinc electrodeposition can be represented as follows:

(3) $$Zn^{2+}+e^{-}\;\to Zn^{+}\left(\mathrm{rate}-\mathrm{determining}\;\mathrm{step}\right)$$

(4) $$Zn^{+}+e^{-}\to Zn^{0}\left(\mathrm{fastest}\;\mathrm{step}\right)$$

In this framework, the conversion of Zn^2^⁺ to Zn⁺ via one‐electron transfer constitutes the rate‐limiting step, significantly influencing the overall kinetics of electrodeposition. These findings are further corroborated by more recent studies conducted by Schmickler and Gileadi et al., which explore the complexities of metal electrodeposition.^[^ ^46^, ^47^ ^]^ Their research indicates that for a multivalent metal ion like Zn^2+^, the strongly solvated divalent Zn^2^⁺ ion exhibits a minimum in the potential of mean force at a greater distance from the surface, where its two primary solvation sheaths remain intact. However, as the ion approaches the surface and sheds its secondary solvation sheath, the potential begins to increase steeply.^[^ ^48^ ^]^

### 2.2 Pitfalls of Tafel Analysis for Zinc Electrodeposition Kinetics

In this section, we discuss the considerations related to the commonly used Tafel analysis method for extracting electron transfer kinetics in zinc metal electrodeposition. It is important to recognize that much of the energy storage community relies on Tafel plots from linear polarization curves to determine kinetic parameters, rather than using more rigorous approaches such as fitting experimental data to electron‐transfer kinetic models as shown earlier. This is not only true to AZMB community but the overall battery community in general. Here, we highlight the potential pitfalls of this approach if not applied cautiously and propose strategies to mitigate these challenges, ensuring more accurate and reliable analysis.

In general, Tafel behavior is observed at sufficiently large overpotentials, which allows for the simplification of one of the terms in the brackets of Equation (1). For example, at large negative overpotentials, Equation (1) simplifies to Equation (5) which in turn leads to Equation (6). This high‐overpotential approximation is commonly utilized in the battery community to determine the exchange current values, which are often assosiated with the long‐term stability of the anode.

(5) $$i=i_{0}e^{-\alpha f\eta }$$

(6) $$\eta =\frac{RT}{\alpha F}\;ln\;i_{0}-\frac{RT}{\alpha F}ln\;i$$

**Figure 2:**
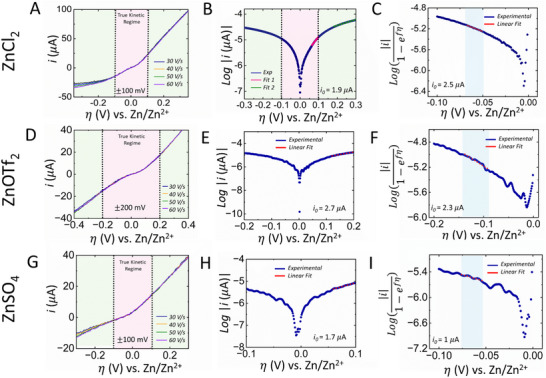
a) Kinetic regime of fast‐scan cyclic voltammogram, b) Tafel plot corresponding to the kinetic regime, and c) Allen‐Hickling plots for the kinetic regime. (a), (b), and (c) show data for 1 M ZnCl_2_ as the electrolyte. In a similar fashion, d), e), and f) show the respective data for 1 M ZnOTf_2_, and g), h), and i) respective data for 1 M ZnSO_4_.

A plot of η versus *ln i* serves as a convenient method to deduce the value of *i*_0_​ from the intercept and α from the slope of the linear fit. The key consideration is that the Tafel method, whether for the cathodic or anodic branch at sufficiently large overpotentials, will only hold true when the reverse reaction (e.g., the anodic process during net reduction, or vice versa) contributes to less than 1% of the total current.^[^ ^31^, ^49^ ^]^ A simple calculation, using the ratio of anodic to cathodic currents, reveals that this condition is met when |η| >118 mV at 25 °C. This consideration is crucial for practical applications like electrodeposition. If the electrode kinetics are sufficiently fast, the system may reach a mass‐transfer‐limited current before such an overpotential is established. As a result, a true kinetic regime, where accurate Tafel analysis can be performed, may not be achieved due to the influence of mass‐transfer limitations. Nevertheless, one could still carry out Tafel analysis in this region (which is usually done in the community), but the extracted values may be inaccurate and could lead to erroneous conclusions. To further illustrate this concept, consider **Figure** **2**a, which highlights the kinetic regime of the cyclic voltammograms presented in Figure 1c for 1 M ZnCl_2_ as the electrolyte. It is evident that the current transients overlap only within a narrow overpotential (±0.1 V) range near the crossover potential, indicating the absence of mass‐transfer effects in this region. Beyond this range, particularly at more negative cathodic overpotentials, the current transients no longer overlap, indicating the onset of mass‐transfer limitations. It is important to note that any analysis in this region would be influenced by mass‐transfer effects, as demonstrated by the green linear fit in Figure 2b. Using this linear fit, the obtained *i*_0_ value is 12.8 µA, which is significantly higher than the values derived from both BV and MH models with *n* equals 1, as shown in Table 1. In contrast, if similar analysis is performed within the kinetic regime, despite being under 118 mV, the resulting *i*_0_ value is 1.9 µA. While this value is closer to that expected for a one‐electron transfer, conducting the analysis under these conditions is fundamentally incorrect. This is because the current contribution from the back reactions exceeds 1%, indicating that Equation (6) is not applicable in this case. Thus, any kinetic parameters derived from such an analysis may lead to misleading conclusions regarding the electron transfer process.

It is imperative to understand that true Tafel behavior is only observed when the electron transfer kinetics are entirely irreversible, which is not the case for zinc electrodeposition in 1 M ZnCl_2_ as the electrolyte, where zinc electrodeposition falls into the category of “quasi‐reversible” electrode processes.^[^ ^50^, ^51^ ^]^ This is because both anodic and cathodic processes contribute significantly to the measured currents within the overpotential range where mass transfer effects are negligible. Under these conditions, Allen and Hickling (A‐H) proposed an alternative method for probing electron transfer kinetics at low overpotentials.^[^ ^33^ ^]^ Essentially, Equation (1) can be reformulated as Equations (7) and (8):

(7) $$i=i_{0}e^{-\alpha f\eta }\left(1-e^{f\eta }\right)$$

(8) $$Log\;\;\frac{i}{1-e^{f\eta }}=Log\;i_{0}-\frac{\alpha F\eta }{2.3RT}$$

Based on Equation (8) a plot of $Log\;\frac{i}{1-e^{f\eta }}\;$ versus η yields an intercept of *Log* *i*_0_ and a slope $-\frac{\alpha F}{2.3RT}$. A similar plot for the data in the kinetic regime of Figure 2a is shown in Figure 2c for 1 M ZnCl_2_. Using the linear fit shown by the dotted red line, we were able to conveniently determine the exchange current, calculated to be 2.5 µA, and the slope of the fit yielded a transfer coefficient of 0.42. These values are in close agreement with the fitting parameters presented in Table 1 for a one‐electron transfer using both the BV and MH models in 1 M ZnCl_2_ as the electrolyte. Another set of data with BV, MH, Allen‐Hickling, and Tafel analyses for 1 M ZnCl_2_ electrolyte has been shown in Figure S1 to ensure reproducibity, where the results observed were similar.

Considering the quasi‐reversible kinetics observed for zinc electroplating in 1 M ZnCl_2_, it is intriguing to explore other commonly used counter anions, such as ZnSO_4_ and Zn(OTf)_2_, in AZMBs. A similar analysis was performed at 1 M concentration for these anions, revealing that kinetic reversibility varies with the choice of counter anion (see Figures S2 and S3). Notably, for ZnOTf_2_, as shown in Figure 2d, the kinetic regime spans ∼±200 mV, indicating that Tafel analysis is applicable. Interestingly, both Tafel and Allen‐Hickling analyses yield comparable exchange current values of 2.7 and 2.3 µA, respectively. For the case of ZnSO_4_, the kinetic regime was ~±100 mV, similar to ZnCl₂, as shown in Figure 2g. The corresponding Tafel and Allen‐Hickling plots are presented in Figure 2h,i, respectively. The measured *i*_0_ values were 1.7 and 1 µA using the Tafel and Allen‐Hickling models, respectively. This discrepancy is expected due to the quasi‐reversible nature of the system. **Table** **2** summarizes all the measured values for *j*_0_ using the BV, MH, and A–H models, and reorganization energetics for all the counter anions. We admit that the analysis reveals a lack of correlation between the *i*_0_ trend and kinetic regime width. A wider kinetic regime ideally signifies kinetic irreversibility, thus predicting a lower *i*_0_ value. However, ZnOTf_2_, exhibiting the widest kinetic regime (±200 mV), displays an intermediate *i*_0_ value relative to ZnCl_2_ and ZnSO_4_. While this trend is noted, the underlying cause requires further investigation; we hypothesize it stems from the assumptions made regarding the crossover potential equalling the equilibrium potential. This aspect is currently under investigation in our group.

**Table 2:** Kinetic parameters of different zinc electrolytes obtained from AH, BV, MH and Tafel analyses.

Electrolyte	Width of Kinetic Region (mV)	Exchange Current Density, *j*_0_ (*A*/*cm* ^2^) (AH model)	Exchange Current Density, *j*_0_ (*A*/*cm* ^2^) (Tafel model)	Exchange Current Density, *j*_0_ (*A*/*cm* ^2^) (BV model)	Exchange Current Density, *j*_0_ (*A*/*cm* ^2^) (MH model)	Solvent Reorganization Energy, λ (eV) (MH model)
ZnCl_2_	100	0.46	0.34	0.35	0.39	0.53
ZnOTf_2_	200	0.42	0.50	0.08	0.23	0.29
ZnSO_4_	100	0.20	0.32	0.19	0.24	0.32

**Figure 3:**
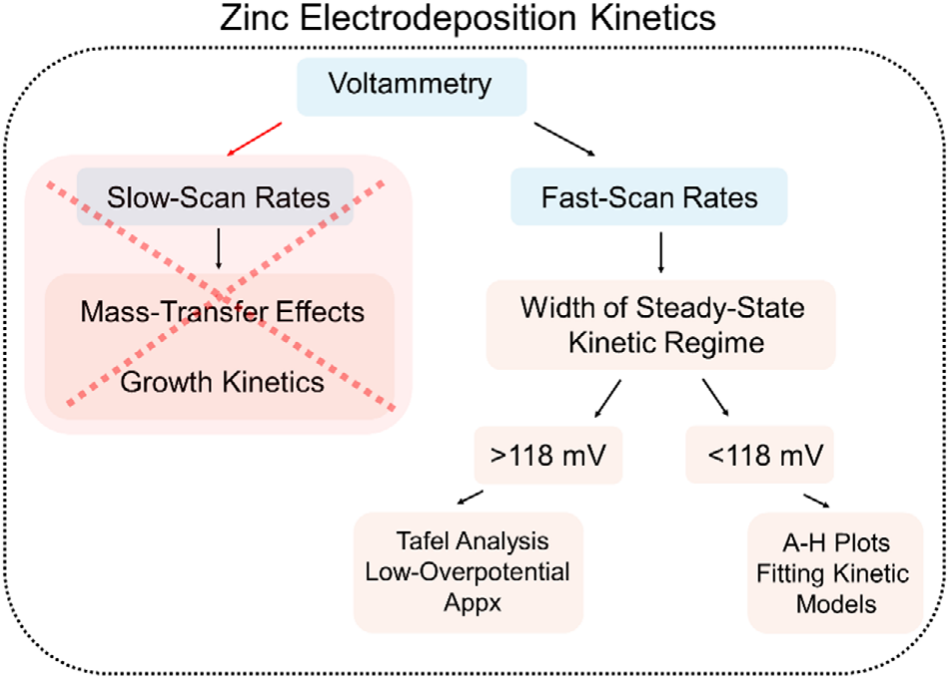
Systematic flow of analysis for analyzing electrodeposition kinetics of zinc electrodeposition.

Based on the discussion, we have outlined the overall flow of analysis in **Figure** **3**. Fast scan voltammetry is crucial for studying the kinetics of zinc electrodeposition due to the presence of a steady‐state kinetic regime. The width of this kinetic regime provides insights into the kinetic reversibility of the electrodeposition energetics. When the regime width exceeds 118 mV on both anodic and cathodic counterparts, Tafel analysis, along with the low‐overpotential approximation of the BV model and fitting the actual models to experimental data, is feasible.^[^ ^31^ ^]^ In contrast, when the regime width is less than 118 mV, A‐H plots and fitting kinetic models offer the most accurate approach to probe electrodeposition kinetics.^[^ ^33^ ^]^ It is important to note that all the analysis tools used for widths less than 118 mV can also be applied to the other case. Overall, A‐H plots can be used universally across all types of steady‐state kinetic regimes, regardless of the width of the regime. Therefore, Allen‐Hickling fit may serve as a more practical method for determining the kinetics of electrodeposition compared to the commonly used Tafel analysis, which is routinely used in the AZMB community. This is because, in Equation (8) we account for the contributions from back reactions at both positive and negative overpotentials in the kinetic regimes, an important factor that is neglected in Tafel analysis. Moreover, another advantage of the A‐H approach over fitting experimental data using the BV or MH models is that it allows for the deduction of the transfer coefficient (α) rather than assuming its value. Considering the findings presented, we suggest that the AZMB community should adopt the use of Equations (7) and (8) as proposed by Allen and Hickling, for deducing kinetic parameters, particularly in the case of quasi‐reversible electrodeposition of zinc, rather than relying on commonly used Tafel analysis.

We emphasize the broad applicability of the presented methodology for advancing research in AZMBs. This approach facilitates efficient screening of electrolyte formulations, additives, and other system variables. However, we acknowledge that the reliance on UMEs may limit its adoption due to their niche usage within the broader AZMB research community. UMEs enable access to a true kinetic regime by supporting fast scan rates, as demonstrated in our previous studies, making them ideal for precise kinetic analysis.^[^ ^4^, ^5^, ^15^ ^]^ To broaden the utility of this methodology, we recommend adaptations for conventional three‐electrode setups with macroelectrodes. Researchers must carefully monitor the deposited charge during voltammetry and ensure that scan rate‐dependent polarization curves establish a steady‐state kinetic regime before conducting kinetic analyses. This precaution is essential to avoid the influence of mass‐transfer effects and maintain the accuracy and reliability of the results. Once a true kinetic regime is established, the Allen‐Hickling approach can be employed to obtain reliable kinetic parameters irrespective of the width of the kinetic regime.

### 2.3 Implication of Accurate Kinetic Analysis on Coin Cell Performance

The implications of this work extend beyond the experimental zinc battery community. Within the community, there is unprecedented disagreement regarding the reporting of important kinetic parameters. These parameters are critical for developing accurate models to understand and predict electrochemical processes.^[^ ^22^, ^23^, ^24^, ^25^, ^26^, ^27^, ^28^, ^29^, ^30^ ^]^ An accurate value for the exchange current is especially crucial when modeling and simulating electrodeposition of zinc using these kinetic parameters. As detailed in this work, utilizing this platform allows us to accurately probe these kinetics and obtain reliable values that are not convoluted by mass transfer effects. Now that we have established this approach, we can test theories that correlate coin cell stability from a kinetic perspective. Specifically, a lower *i*_0_ or *j*_0_ is beneficial, as sluggish kinetics lead to reduced concentration polarization, delaying the onset of dendrite formation, as predicted by the Sands' Equation.^[^ ^35^ ^]^ We omit a discussion of mass transfer contributions, as the diffusion coefficients are similar for all counter anions, as shown in Figure S4.

**Figure 4:**
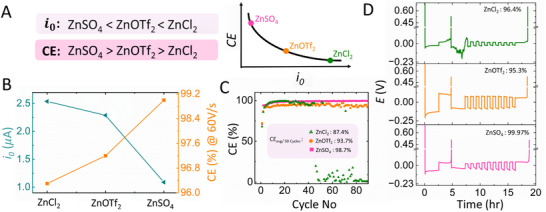
a) Schematic representation of the correlation between exchange current, *i*_0_ and coulombic efficiency for different zinc electrolytes. b) Double y‐axis plot showing how the coulombic efficiency obtained from fast scan voltammetry changes with *i*_0_ of different zinc electrolytes. c) Coulombic efficiency of Cu|Zn asymmetric cell cycling at a current density of 1 mA cm^−^^2^ and a capacity of 0.5 mAh cm^−^^2^ for different zinc electrolytes. d) Aurbach protocol for evaluating Zn stripping/plating Coulombic efficiency (CE) with varying zinc electrolytes in Cu|Zn asymmetric cell.

To begin, we observe a trend in *i*_0_ values, as shown in **Figure** **4**a, where ZnSO_4_ exhibits the lowest *i*_0_, followed by Zn(OTf)_2_ and ZnCl_2_. Based on the Sands model, we hypothesize that ZnSO_4_ should outperform both other electrolytes in terms of long‐term stability and metrics like CE values (See Figure 4a). It is important to note that this trend in *i*_0_ with appropriate current magnitudes will only be observed if accurate kinetic parameter measurements are obtained. If conventional voltammetry is performed at typical scan rates in the range of mV/s, a similar trend is observed but with a significant magnitude difference in absolute values, which increases the chance of error in predicting the battery performance. This data is presented in Figure S5.

Firstly, it was observed that the *i*_0_ value closely agreed with the CE values calculated using fast scan voltammetry at a scan rate of 60 Vs^−1^. This data is shown in Figure 4b. The plots used for calculating CE are shown in Figure S6. A scan rate of 60 Vs^−1^ was chosen because all counter anions exhibited the presence of a kinetic regime at this rate, ensuring that no mass transfer effects were involved. Evidently, a lower value of *i*_0_ correlated with a higher CE value, suggesting that 1 M ZnSO_4_ should outperform 1 M Zn(OTf)_2_, followed by 1 M ZnCl_2_, in terms of long‐term coin cell stability. This was validated using Cu|Zn asymmetric cell cycling at a current density of 1 mA cm^−^^2^ and a capacity of 0.5 mAh cm^−^^2^. It was found that ZnSO_4_ outperforms the other counter anions, with an average CE value of 98.75% over 50 cycles, while Zn(OTf)_2_ showed 93.7% and ZnCl_2_ 87.4%. Additionally, the ZnCl_2_ cell failed only after 50 cycles, and Zn(OTf)_2_ exhibited unstable CE data, whereas ZnSO_4_ showed stable performance over 450 cycles (see Figure S7). Furthermore, these asymmetric cells were tested using the Aurbach protocol,^[^ ^41^, ^52^, ^53^ ^]^ as shown in Figure 4d. The details of the cycling conditions and CE calculation method are provided in the SI. Similar to the asymmetric cell stability data, the same trend was observed, where ZnSO_4_ outperforms the other counter anions with a CE value of 99.7%, while Zn(OTf)_2_ showed 95.3% and ZnCl_2_ 96.4%. Although a higher CE was observed for ZnCl_2_ compared to Zn(OTf)_2_, the voltage profile for ZnCl_2_ exhibited erratic fluctuations. This data clearly supports the correlation presented in Figure 4a, validating the finding that lower, accurately measured *i*_0_ values lead to higher CE values. Overall, we clearly illustrate how obtaining accurate values of *i*_0_ is crucial by using the correct analytical methodology, enabling us to understand the relative trends in the long‐term stability of AZMBs. This study underscores the need for rigorous kinetic models to accurately characterize zinc electrodeposition processes, facilitating the standardization and development of next‐generation AZMBs.

## 3 Conclusion

This study establishes a robust methodology for accurately determining *i*_0_ or *j*_0_ for zinc electrodeposition, addressing inconsistencies in kinetic parameter reporting within the field. By employing fast scan voltammetry on W UMEs, we successfully decoupled mass transfer effects and identified a one‐electron rate‐limiting step in the two‐electron zinc deposition process. Our findings highlight the limitations of conventional Tafel analysis and demonstrate the reliability of the Allen‐Hickling approach for kinetic measurements. The consistency of our results across ZnSO_4_, Zn(OTf)_2_, and ZnCl_2_ underscores the broad applicability of this method, revealing a direct correlation between lower *i*_0_ values and improved battery performance. Coin cell studies confirm that electrolytes with lower *i*_0_ exhibit higher CE, aligning with predictions from Sands’ model. This work provides the first systematic kinetic investigation of counter anions in AZMB community, offering critical insights into how electrolyte composition influences charge transfer kinetics. By linking fundamental electrochemical kinetics to practical battery stability, our findings establish a new framework for optimizing electrolyte formulations, ultimately advancing the development of more efficient and durable AZMBs.

## Conflict of Interest

The authors declare no conflict of interest.

## Author Contributions

A.R. and M.A.F. contributed equally to this work. The authors reserve the rights to list the two names in any order. All the experiments were performed by A.R., M.A.F., and J.E.D. supervised all aspects of the work. All authors have agreed to the final version of the manuscript.

## Supporting information



Supporting Information

## Data Availability

The data that support the findings of this study are available from the corresponding author upon reasonable request.
