# Supplementary material for: Probing Kinetics Beyond Tafel Unlocks Highly Accurate Exchange Currents for Aqueous Zinc Metal Batteries
Source: Small Methods. 2025 Apr 24;10(2):2500508. doi: 10.1002/smtd.202500508 (PMC12825348; doi:10.1002/smtd.202500508)
Supplement: Supplementary file 1 — Supporting Information [file SMTD-10-2500508-s001.docx]

**Supplementary**

**Information**

**Probing Kinetics Beyond Tafel Unlocks Highly Accurate Exchange Currents for Aqueous Zinc Metal Batteries**

Ashutosh Rana^a,£^, Md. Arif Faisal^a,£^, and Jeffrey E. Dick^a,b^*

^a^ Department of Chemistry, Purdue University, West Lafayette, IN, 47907, USA

^b^ Elmore Family School of Electrical and Computer Engineering, Purdue University, West Lafayette, IN, 47907, USA

**Corresponding Author:**

Jeffrey E. Dick ([jdick@purdue.edu](mailto:jdick@purdue.edu))

**Experimental Section**

1. **Materials**

Zinc Chloride (ZnCl_2_, reagent grade), Zinc Triflate (ZnOTf_2_, reagent grade), Zinc Sulfate (ZnSO_4_, reagent grade), Hexaammineruthenium (III) chloride, and Potassium Chloride (KCl, reagent grade) were bought from Sigma Aldrich. Zinc foil (99.99%) was procured from MTI Supplies. 25 µm Tungsten metal wire was bought from Good Fellow. All the chemicals were used without further purification. A Milli-Q ultrapure water production system was utilized to obtain the deionized (DI) water needed to prepare all the aqueous electrolytes.

1. **Preparation of Electrolyte**

The electrolytes were prepared by dissolving 1 mol/L (M) ZnCl_2_ in DI water at room temperature (25 °C). Similarly, 1 M ZnOTf_2_ and 1 M ZnSO_4_ solutions were prepared by dissolving the appropriate amounts of salts in DI water.

1. **Fabrication of Ultramicroelectrode**

The fabrication of ultramicroelectrodes (UMEs) is a precise and multi-step process involving the assembly of three main components: a capillary, a metal wire as the electrode material, and an electrical wire to complete the connection. A crucial aspect of this process is the selection of the capillary, which serves as the structural framework of the UME. Glass is the most widely used material for capillaries because of its versatility and suitability for a broad range of applications, from standard temperature analyses to challenging environments like molten salt studies. The inner diameter (ID) of the capillary is critical to ensure proper sealing with the metal wire. An optimal ID falls between 1.1 and 1.2 mm, striking a balance that allows the wire to fit securely without excessive difficulty during sealing. Additionally, the thickness of the glass must allow it to melt easily during the sealing process while remaining rigid enough to avoid bending. Capillary length also plays an important role; longer capillaries are suitable for probing deeper into samples and reducing ohmic drop in low-conductivity media, whereas shorter ones are ideal for high spatial resolution, reduced background noise, and fast kinetics studies. For most applications, a length of around 50 mm offers a good compromise, particularly for rapid kinetics measurements. The correct selection of the capillary material, dimensions, and thickness is vital because a poorly chosen capillary can compromise the entire fabrication process, leading to issues with sealing and rendering the UME unusable.

The choice of electrode material is equally significant, as it determines the UME’s electrochemical properties. While the type of material depends on the intended application, the diameter of the metal wire is especially important. The critical diameter for ensuring only radial diffusion at the electrode surface is 25 µm, and wires exceeding this threshold may not produce the desired diffusion profile.^[1–3]^ Once the materials are prepared, the first step in fabrication involves sealing one end of the capillary with a propane torch. This step requires steady rotation of the capillary in the flame to prevent bending of the glass, creating a smooth, pocket-like structure at the sealed end. This pocket is critical for the subsequent step of threading the metal wire. The wire is carefully inserted into the capillary using sharp tools to ensure a clean, smooth tip and to avoid introducing dust particles, which can interfere with the process. The length of the metal wire must be sufficient to connect with the electrical wire later but must not extend through the other end of the capillary. Gentle tapping ensures the wire settles into the sealed pocket, where it gains stability, allowing for successful vacuum sealing.

Vacuum sealing is the most crucial step in UME fabrication, as it ensures a smooth, bubble-free seal around the metal wire. During this step, the capillary is connected to a vacuum pump to eliminate trapped air while the glass is heated uniformly using a heating coil. Consistent heating prevents bubble formation and ensures the seal around the wire is homogenous and strong. Careful temperature control is essential to avoid melting the metal wire during this process. After vacuum sealing, the electrical wire is connected to the metal wire to complete the UME’s circuitry. This is achieved by coating the electrical wire with gallium, which ensures a secure connection when inserted into the capillary. A rubber tube is used to fix the electrical wire in place, ensuring the connection remains stable during use.

The final step involves exposing and polishing the metal wire tip to prepare the UME for use. Coarse sandpaper is initially employed to remove excess glass from the sealed end of the capillary, followed by finer grades of sandpaper to smooth the surface. Polishing is done in a traditional “8” motion to achieve a uniform finish. Once the wire tip is exposed, it is further refined using alumina slurry on a polishing pad, resulting in a clean, defect-free electrode surface. This ensures the UME achieves optimal performance during electrochemical measurements.

The successful fabrication of UMEs depends on the precise selection of materials and adherence to each step of the process. The capillary’s dimensions and material, the electrode material's properties, and the execution of sealing, connection, and polishing steps all contribute to the quality of the final product. This comprehensive approach ensures the production of high-performance UMEs, which are essential tools in a variety of electrochemical research applications, including high-resolution spatial measurements and studies of rapid kinetics.

1. **Method of Testing the W Ultramicroelectrode**

Employing a two-electrode setup instead of the conventionally used three-electrode setup is not problematic when working with a UME as the working electrode. The current passed through the system for measurements is typically very small, thereby allowing the potential drop at the working electrode to be referenced against a stable potential provided by the Ag/AgCl in 1 M KCl reference/counter electrode. All the electrochemical measurements were conducted using 1 M ZnCl_2_ as the electrolyte. Prior to conducting any zinc electrodeposition on the W UME, a cyclic voltammogram was recorded using a well-behaved outer-sphere redox molecule (Hexaammineruthenium (III) chloride, RHT) to ensure the observation of a steady-state current, as is typical for a UME. **Fig. 1(b)** illustrates a sigmoid-shaped cyclic voltammogram obtained using the same experimental setup as described earlier, with 5 mM RHT as the electrolyte. The presence of low capacitance and steady-state currents confirms the accurate fabrication of the UME.

1. **Electrochemical Measurements**

A CHI6284E electrochemical workstation was used for all electrochemical measurements. As mentioned in the main publication, a two-electrode setup was used for CV measurements.

1. **Aurbach Protocol for Coin Cell Performance**^[4]^

Substrate-related factors such as lattice mismatch, alloy formation, and interphase interactions can impact Coulombic efficiency (CE) measurements in various coin cell configurations.^[5,6]^ To isolate the effect of different zinc electrolytes and mitigate these substrate influences, we employed the galvanostatic method described by Adams et al. and Xu et al., as illustrated in **Fig. 4(d)**.^[6,7]^ Specifically, to ensure consistency across all concentrations, an initial conditioning cycle was performed, during which 5 mAh cm⁻² of Zn was deposited onto and removed from the Cu working electrode at a current density of 2 mA/cm², with a cutoff voltage of +0.5 V. Following this step, an additional 5 mAh cm⁻² of Zn was plated onto the Cu (acting as a reservoir), providing a controlled and finite Zn source for accurate CE assessment (Q_r_) at the same current density. The cycling protocol then involved repeated deposition and stripping of a fixed 1 mAh cm⁻² (Q_c_) for nine cycles, followed by complete Zn removal to +0.5 V versus Zn/Zn²⁺ to ensure all extractable Zn, including the initial reservoir (Q_s_), was stripped. The CE was determined using Equation 1.

$CE=\frac{{9Q}_{c}+Q_{s}}{{9Q}_{c}+Q_{r}}$………………………………………………(1)

**
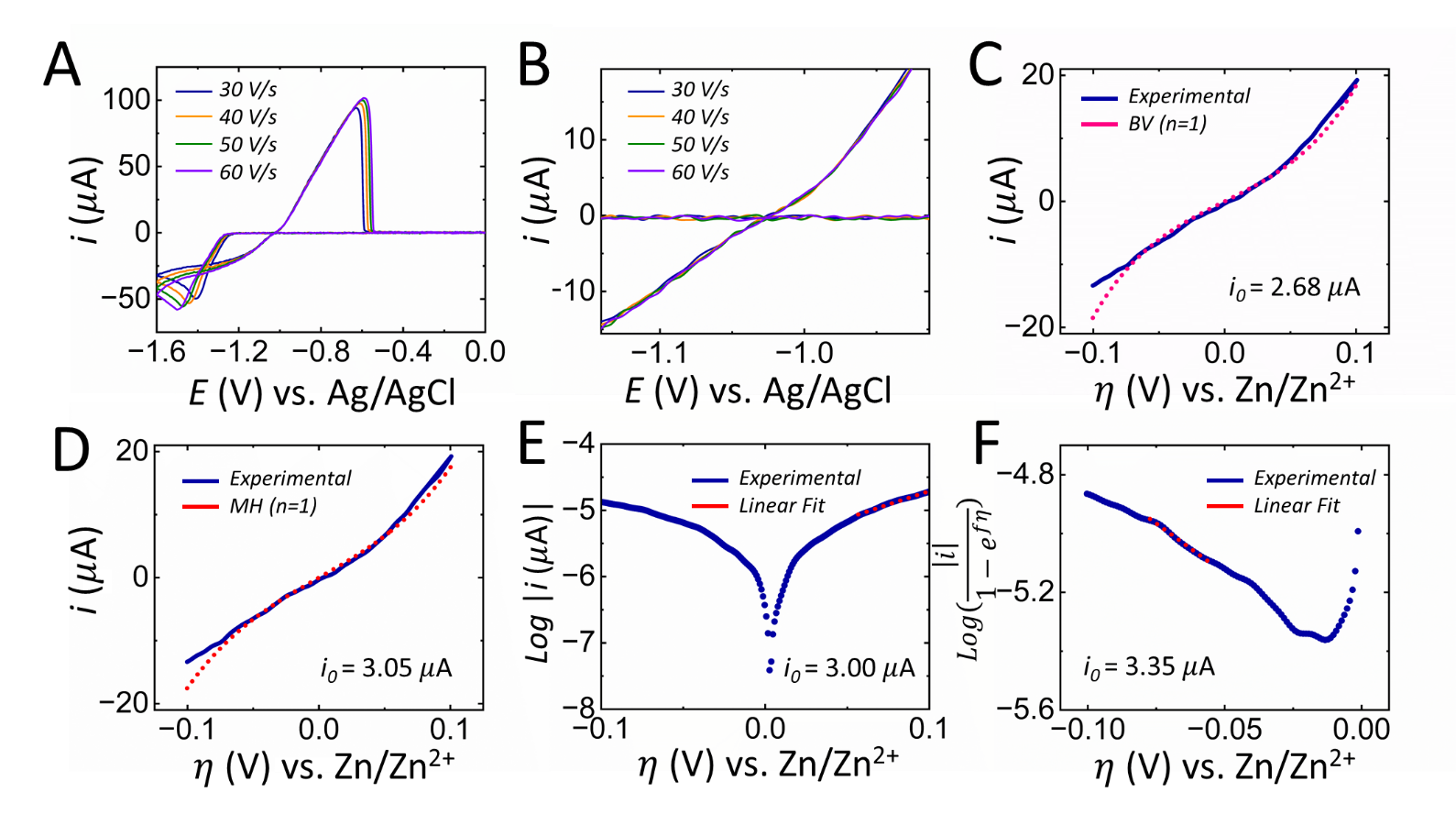
Fig. S1** (a) Fast-scan cyclic voltammograms of ZnCl_2_ electrolyte, recorded for the electrodeposition of zinc on a tungsten UME at different scan rates. (b) Zoomed in image of the kinetic region where the voltammograms are independent of scan rates. (c) Butler-Volmer fitting of the kinetic region, allowing us to calculate the exchange current. (d) Marcus-Hush fitting of the kinetic region for the determining the exchange current and solvent reorganization energy. (e) Tafel plot obtained from the kinetic region. (f) Allen-Hickling plot of the kinetic region for the accurate calculation of exchange current.

**
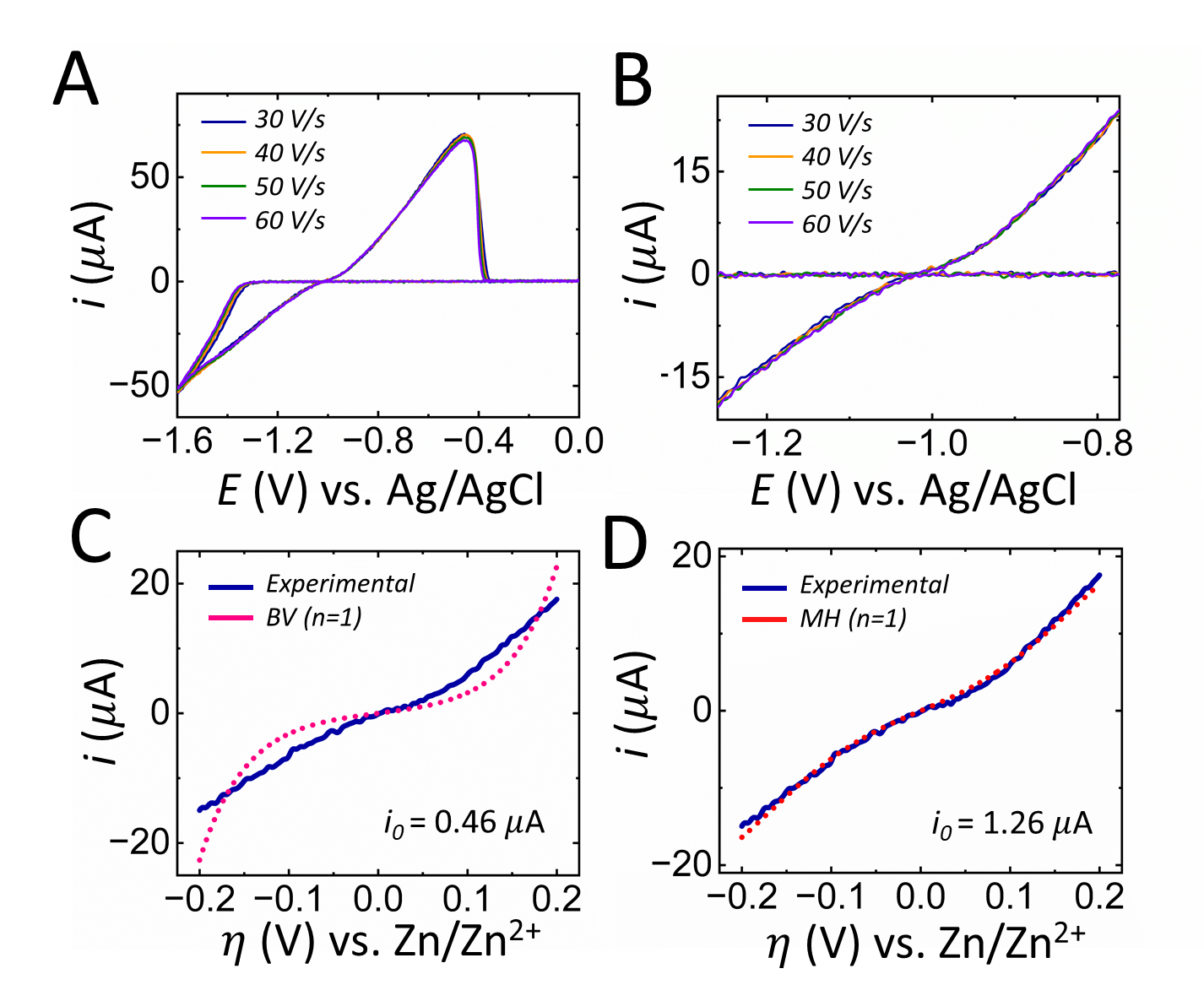
Fig. S2** (a) Fast-scan cyclic voltammograms of ZnOTf_2_ electrolyte, recorded for the electrodeposition of zinc on a tungsten UME at different scan rates. (b) Zoomed in image of the kinetic region where the voltammograms are independent of scan rates. (c) Butler-Volmer fitting of the kinetic region, allowing us to calculate the exchange current. (d) Marcus-Hush fitting of the kinetic region for the determining the exchange current and solvent reorganization energy.

**
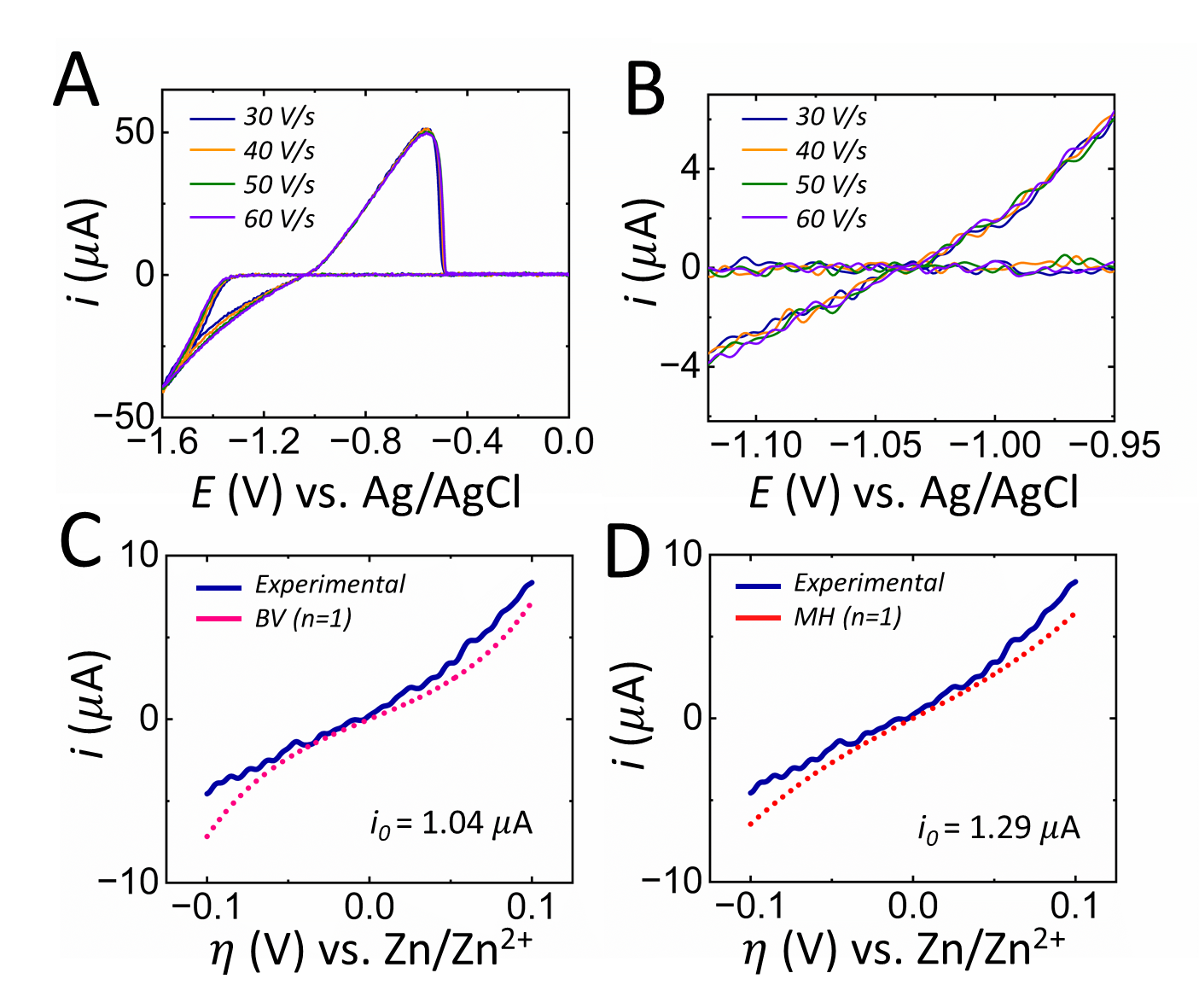
Fig. S3** (a) Fast-scan cyclic voltammograms of ZnSO_4_ electrolyte, recorded for the electrodeposition of zinc on a tungsten UME at different scan rates. (b) Zoomed in image of the kinetic region where the voltammograms are independent of scan rates. (c) Butler-Volmer fitting of the kinetic region, allowing us to calculate the exchange current. (d) Marcus-Hush fitting of the kinetic region for the determining the exchange current and solvent reorganization energy.

**
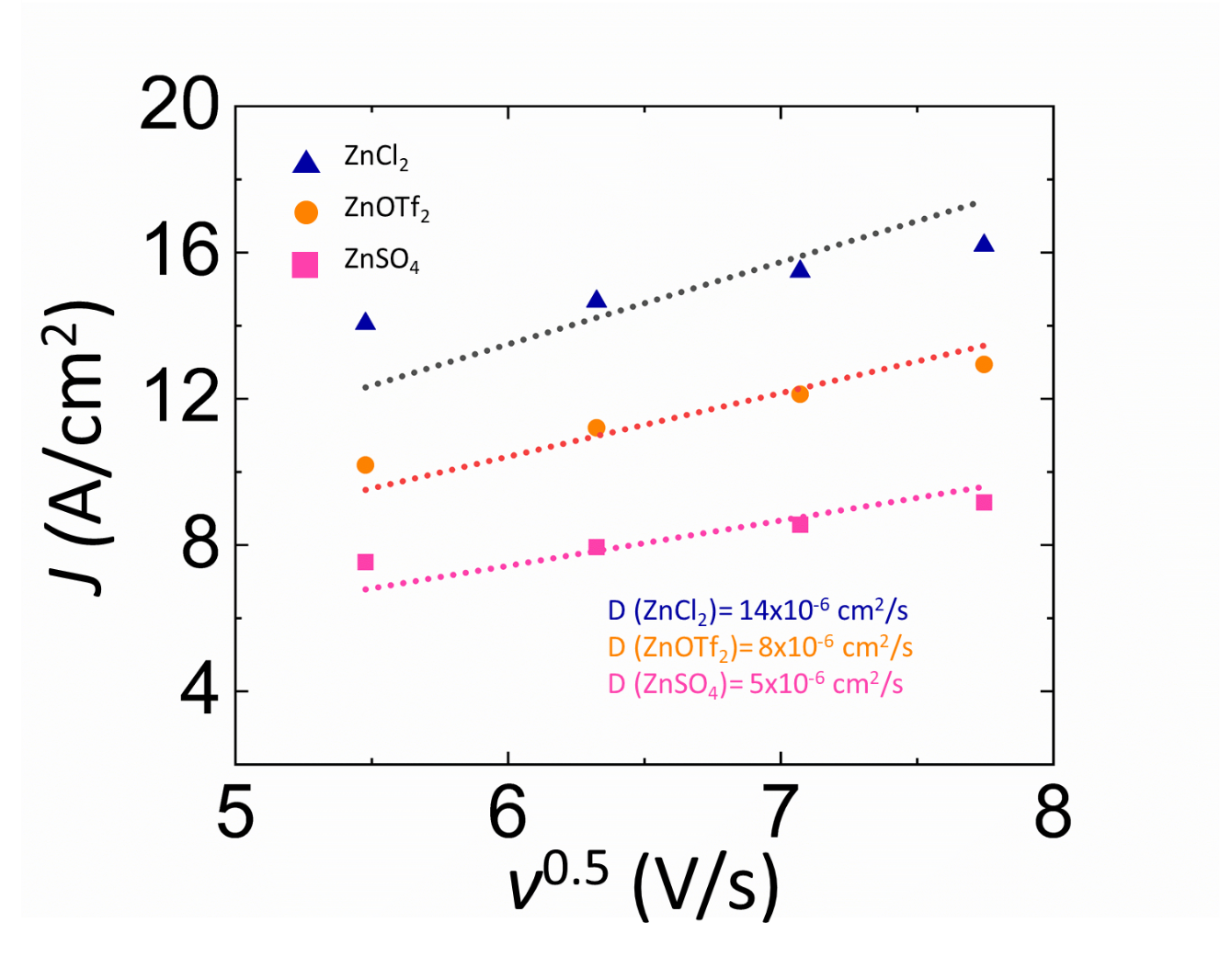
**

**Fig. S4** Diffusion coefficient (*D*) of Zn^2+^ in 1 M ZnCl_2_, ZnOTf_2_ and ZnSO_4_ solutions.

**
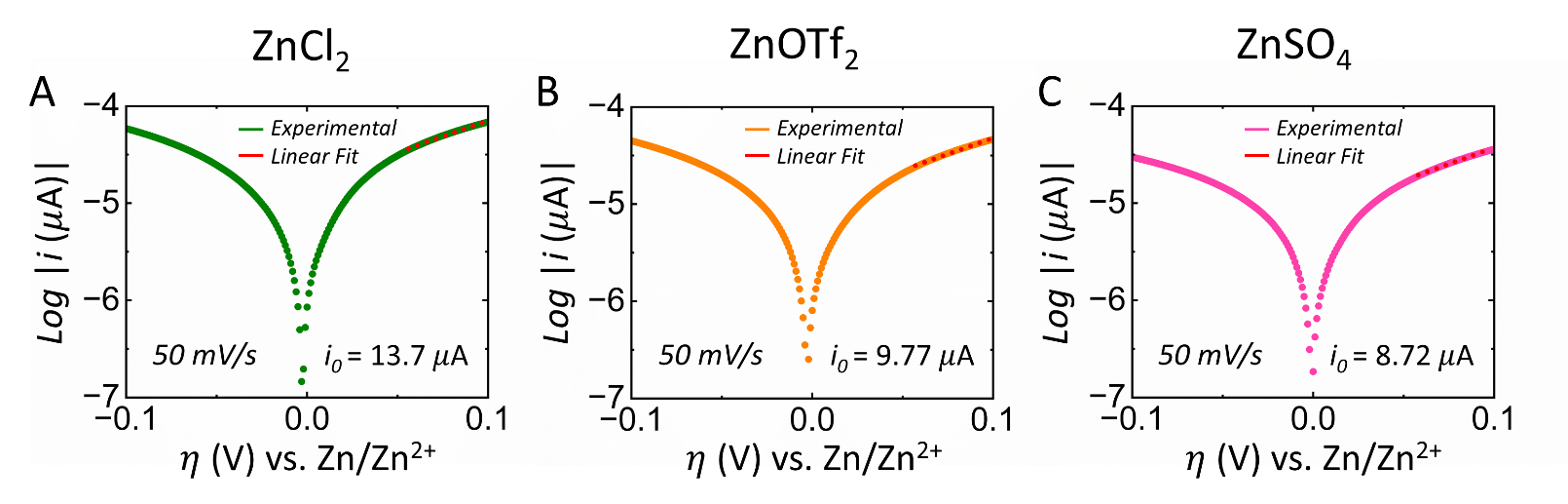
**

**Fig. S5** Tafel analysis for slow scan voltammetry of zinc electrodeposition on tungsten ultramicroelectrode. Since there is no true kinetic region in slow scan voltammetry, the same potential window was taken for all three zinc electrolytes.

**
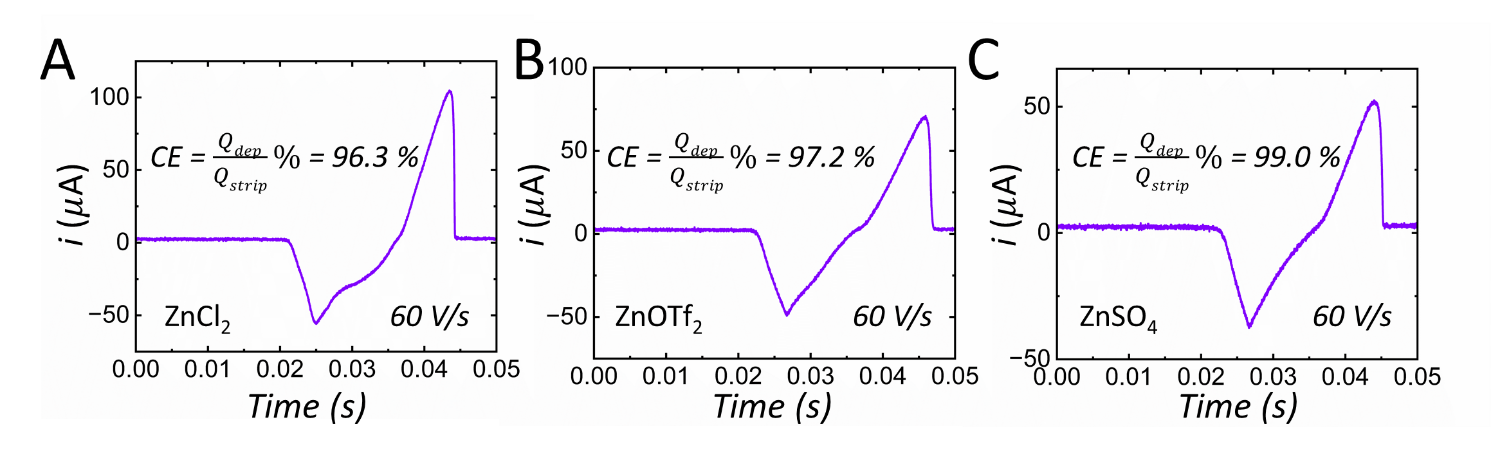
****Fig. S6** Current versus time plots obtained from the FSCVs for different electrolytes, allowing us to determine the coulombic efficiency (CE) for the electrodeposition of zinc.

**
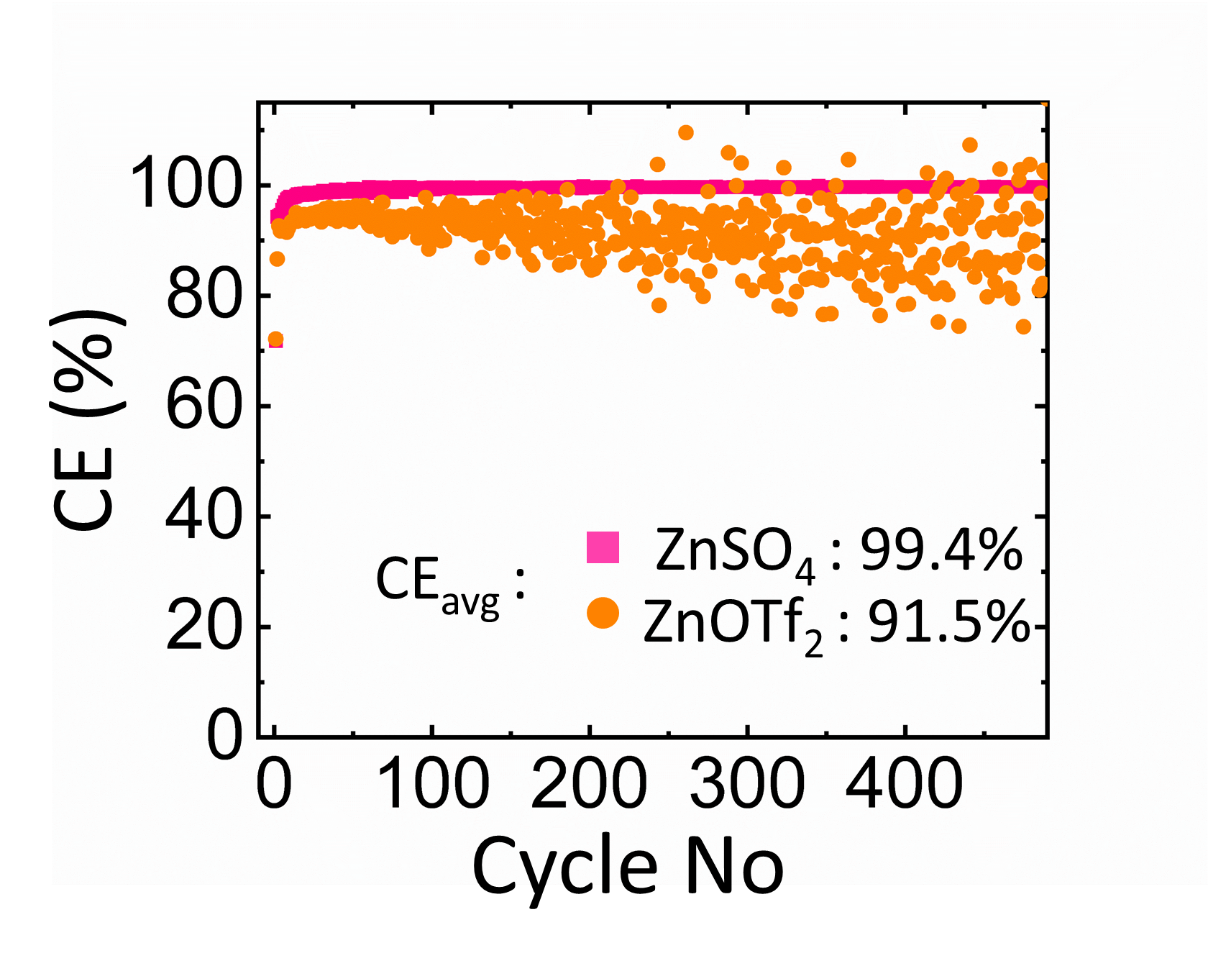
**

**Fig. S7** Coulombic efficiency of Cu|Zn asymmetric cell cycling at a current density of 1 mA/cm² and a capacity of 0.5 mAh/cm² for ZnSO_4_ and ZnOTf_2_.

**References**

[1] A. Bard, L. Faulkner, H. White, *Electrochemical Methods: Fundamentals and Applications*, **2022**.

[2] J. Heinze, *Angewandte Chemie International Edition in English* **1993**, *32*, 1268.

[3] M. A. Faisal, J. E. Dick, *ACS Appl Energy Mater* **2024**, *7*, 10326.

[4] D. Aurbach, I. Weissman, A. Zaban, O. Chusid, *Electrochim Acta* **1994**, *39*, 51.

[5] X. Yu, Z. Li, X. Wu, H. Zhang, Q. Zhao, H. Liang, H. Wang, D. Chao, F. Wang, Y. Qiao, H. Zhou, S. G. Sun, *Joule* **2023**, *7*, 1145.

[6] L. Ma, M. A. Schroeder, O. Borodin, T. P. Pollard, M. S. Ding, C. Wang, K. Xu, *Nat Energy* **2020**, *5*, 743.

[7] B. D. Adams, J. Zheng, X. Ren, W. Xu, J. Zhang, *Adv Energy Mater* **2018**, *8*, DOI 10.1002/aenm.201702097.
